# Diffusion weighted MR imaging in the differential diagnosis of haemangiomas and metastases of the liver

**DOI:** 10.2478/v10019-010-0001-4

**Published:** 2010-03-18

**Authors:** Nagihan Inan, Furkan Kilinc, Tahsin Sarisoy, Sevtap Gumustas, Gur Akansel, Ali Demirci

**Affiliations:** Department of Radiology, University of Kocaeli, Kocaeli, Turkey

**Keywords:** liver, haemangioma, metastasis, magnetic resonance imaging, diffusion-weighted imaging, apparent diffusion coefficient

## Abstract

**Background:**

The purpose of the study was to evaluate the value of diffusion-weighted imaging in the differential diagnosis of haemangiomas from metastases of the liver.

**Patients and methods.:**

We analyzed 69 lesions in 38 patients (33 haemangiomas; 36 metastases) in the retrospective study. Diffusion-weighted imaging was performed using a breath-hold single-shot echo-planar spin echo sequence with three b factors (0, 500 and 1000 sec/mm^2^), and apparent diffusion coefficients (ADCs) were calculated. For the quantitative evaluation, signal intensity of the lesions, lesion-to-liver signal intensity ratios, ADC of the lesions, and lesion-to-liver ADC ratios were compared between the groups. The statistical significance was determined by student’s-t test.

**Results:**

With the b factor 500 sec/mm^2^, no statistical significance was achieved (p>0.05). With the b factor of 1000 sec/mm^2^, both the signal intensity and lesion-to-liver signal intensity ratio of the metastases were significantly higher than those for haemangiomas (p<0.001). The cut-off value at 2.6 yielded a sensitivity of 86% and a specificity of 82% for the lesion-to-liver signal intensity ratio. The ADC, and lesion-to-liver ADC ratio of the metastases were significantly lower than those of haemangiomas (p<0.001). With cut-off value of 1.7, ADC ratio had a sensitivity of 88% and a specificity of 72% for ADC lesion/liver.

**Conclusions:**

Diffusion-weighted imaging with high b value may help in the differential diagnosis of metastases from haemangiomas of the liver.

## Introduction

A liver lesion detected in a patient with the known malignant disease requires a further assessment, as the liver is common site for metastatic spread and haemangiomas are encountered in about 7–20% of the population.^1^ The radiologic imaging plays a critical role in the differential diagnosis of these lesions. On postcontrast computed tomography (CT) and magnetic resonance (MR) images, most haemangiomas have a typical enhancement. However, atypical haemangiomas may imitate metastases. The differential diagnosis of these lesions is essential to determine the therapy.^2^ A variety of radiologic imaging is currently available for the clinical use in these cases.^3^ CT arterioportography has been widely used in the differential diagnosis, however, this technique is invasive and the results are not always reliable.^4^ Over the years, the success rates have increased with the development of new MR contrast agents such as superparamagnetic iron oxide (SPIO).^3^ However, we may encounter some problems in interpreting SPIO-enhanced MR images because of the difficulty in differentiating thin vessels, small cysts, haemangiomas, and metastases.^5^ Therefore, a non-invasive method is required in the diagnosis of such lesions.

In this study, we evaluated the contribution of diffusion-weighted (DW) imaging in the differentiation of metastases from haemangiomas, particularly in a patient with the known malignant disease, which poses a challenge in the differential diagnosis.

## Patients and methods

### Patients

Our retrospective data were obtained in a 14-month period (September 2007 to October 2008). During this period 63 patients were referred for MR imaging of the liver for the following indications: suspected haemangioma or metastatic liver mass based on the findings of other imaging modalities and the evaluation for metastases in patients with known primary cancer. However, 25 patients were excluded from the study because of size (< 1cm) (n = 11), low image quality of DW images (n = 8), and incomplete characterization of lesions on the follow-up imaging or the histopathologic examination (n = 6). As a consequence, a total of 69 solid lesions with a diameter of at least 1 cm in 38 patients (20 women, 18 men) were included in this study. Of these lesions, 36 (in 15 patients) were metastases and 33 (in 23 patients) were haemangiomas. Fifteen patients had multiple lesions (two metastases in 1 patient, three metastases in 3 patients, five metastases in 1 patient, six metastases in 2 patients, two haemangiomas in 6 patients, three haemangiomas in 2 patients). For subjects with more than six lesions only six largest lesions and one region of hepatic parenchyma were analyzed. Imaging was performed prior to the administration of the neoadjuvant treatment or biopsy.

The diagnosis of all metastases was confirmed histopathologically after MR imaging. For subjects with multiple metastases only one lesion was analyzed histopathologically. Remaining similar radiologic appearing lesions were accepted as metastases because all of them increased in size during the radiologic follow-up (4–10 months). The primary cancer sites in each patient were as follows: 3 colorectal cancers, 2 pancreatic cancers, 1 common bile duct cancer, 3 lung cancers, 2 breast cancers, 1 non-Hodgkin’s lymphoma, 1 neuroblastoma, 1 endometrial cancer, 1 adenoid cystic carcinoma of the appendix. All patients with a tentative radiological diagnosis of haemangiomas showed no change during the clinical and radiological follow up (US every 3 months for 9–14 months). Five patients with haemangiomas had a primary cancer as following sites: 3 colorectal cancers, 2 breast cancers.

### MR imaging

The study was approved by the institutional review board and the protocol review committee. Since the tests employed were a part of the routine clinical work-up of these patients, the informed consent was not required by the review board.

All patients were examined with a 1.5 Tesla MR scanner (Gyroscan Intera; Philips Medical Systems, Eindhoven, The Netherlands) using a four element phased-array body coil. This system has a maximal gradient strength of 30 mT/m and a slew rate of 150 mT/m/msec. All patients were examined initially with the routine MR imaging protocol for the upper abdomen that included: precontrast axial T1-weighted breath-hold spoiled gradient echo (fast field echo: FFE) with and without fat suppression (TR/TE/FA/NEX:169/4.6/80/1), coronal and axial T2-weighted single shot turbo spin echo (SS-TSE) (TR/TE/NEX/TSE factor: 700/80/1/72), and axial T2-weighted SS-TSE with fat suppression (TR/TE/NEX/TSE factor: 700/80/1/72). Subsequently, 3 series of axial single-shot spin-echo echo-planar (SS-SE-EP) DW images (TR/TE/echo-planar imaging factor: 1000/81/77; sensitizing gradients in x, y, z directions) were acquired using the following b values: 0, 500 and 1000 sec/mm^2^. ADC maps were reconstructed from these images. The fat suppression was performed by using a spectral saturation inversion recovery (SPIR) technique. Subsequently, 0.1 mmol/kg Gd-DTPA (Magnevist, Schering, Germany) was administered as a hand-injected bolus in 5 seconds followed by a rapid flush with 10–20 ml of saline. Five dynamic series and an additional late phase (5^th^ min) image were acquired with a T1-weighted breath-hold FFE (TR/TE/FA:169/4.6/80) sequence. MR imaging, including the DWI, consisted of a multisection acquisition with a slice thickness of 6 mm, an intersection gap of 1mm, and an acquisition matrix of 128x256. The field of view varied between 455 and 500 mm. All sequences were acquired using a partially-parallel imaging acquisition and SENSE reconstruction with a reduction factor (R) of 2. The scan time of the acquisition of each DW imaging series during a single breath-hold was 26 seconds.

### Image analysis

Quantitative measurements were made using a dedicated work-station (Dell Workstation precision 650, View Forum release 3.4 system). SI of the lesions and liver were measured by one of the radiologists (N.I) for each b factor (0, 500 and 1,000 sec/mm^2^) using a region of interest (ROI). The ROI was placed centrally and the size of the ROI was kept as large as possible, covering at least two-thirds of the lesion, yet avoiding the interference from the surrounding liver tissue and major blood vessels. In addition, the ADC maps were created automatically and the mean ADC values of lesions and liver were determined on images with b factor 0 and 1000 sec/mm^2^. The average of three measurements was recorded as the final SI or ADC. SI of the lesions, lesion-to-liver SI ratio (SIR), ADC of the lesions, and lesion-to-liver ADC ratio (ADCR) were calculated.

### Statistical analysis

SI, SIR, ADC, and ADCR were compared between the groups. The fitness of numeric data set to normal distribution was determined by Kolmogorov-Smirnov test. The data were normally distributed; hence the differences in SIs, SIRs, ADCs, and ADCRs were analyzed by the student-t test. A p value of less than 0.05 was considered statistically significant. To evaluate the diagnostic performance of the quantitative tests (SIR and ADCR) for differentiating metastases from haemangiomas and to describe the sensitivity and specificity of the tests, the receiver operating characteristic (ROC) analysis was performed. The areas and standard errors for each ROC curve were calculated by the method described by Metz.^6^ The area under the ROC curve reflects the performance of the tests. The optimum cut-off point was determined as the value that best discriminates between the two groups in terms of maximum sensitivity and minimum number of false-positive results. All statistical analyses were performed using SPSS (Statistical Package for Social Science) software.

## Results

The mean age was 66.9 ± 9.3 years and 45.5 ± 12.5 years for patients with metastases and haemangiomas, respectively. The mean size for metastases and haemangiomas were 44.7 ± 28.4 mm and 38.1 ± 23.2 mm, respectively. 80% of the metastases were found in the right lobe (segment 5 to 8) with the remaining in the left lobe (segments 1 to 4). 69% of the haemangiomas were found in the right lobe (segment 5 to 8) with the remaining in the left lobe (segments 1 to 4).

**FIGURE 1:**
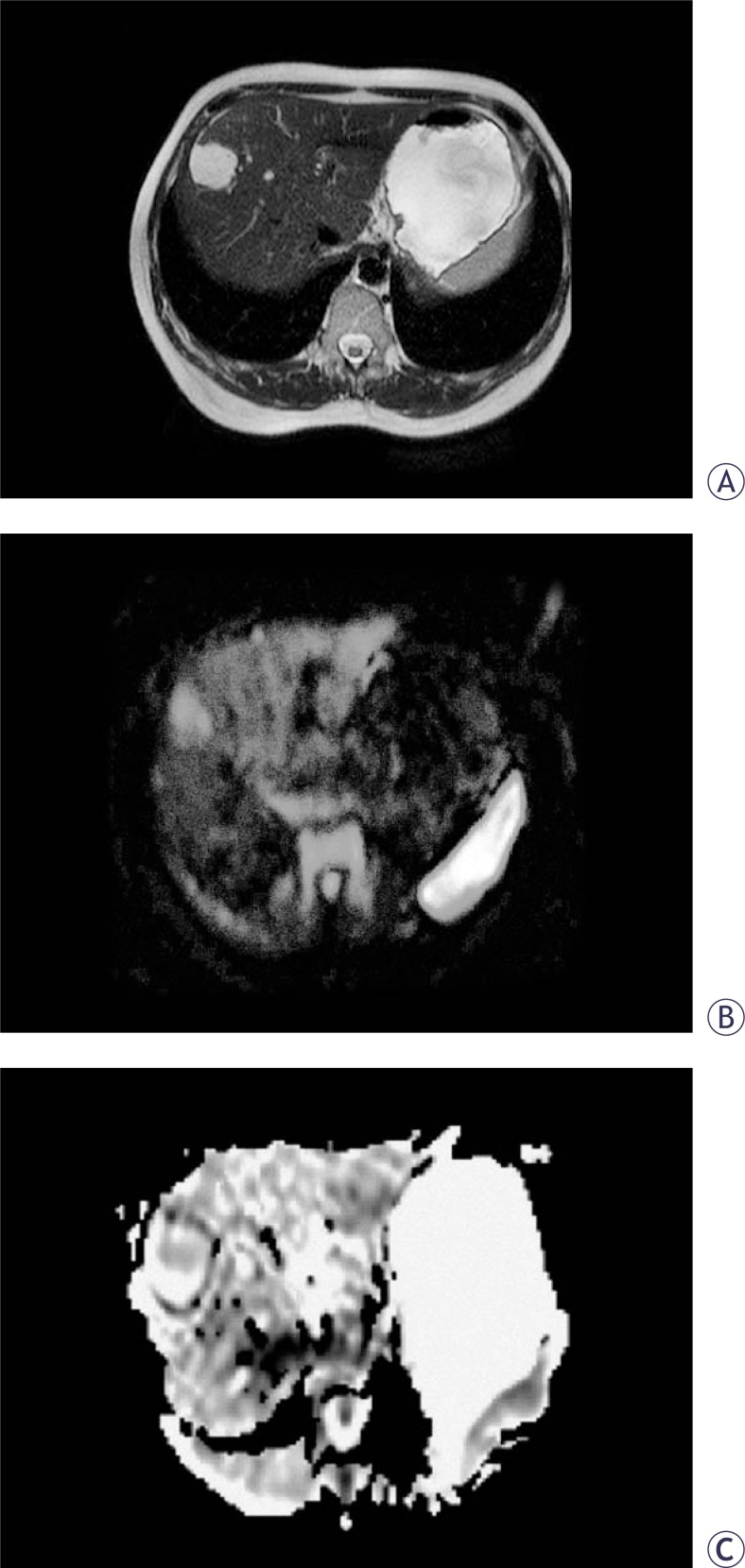
44-year-old woman with a haemangioma of the liver. Axial T2-weighted TSE (A) MR image shows a haemangioma in the right lobe of the liver. This haemangioma appears isointense relative to the liver on the diffusion-weighted image with b factor 1000 sec/mm^2^ (B). ADC map. Lesion-to-liver ADC ratio=1.9 (C).

**FIGURE 2:**
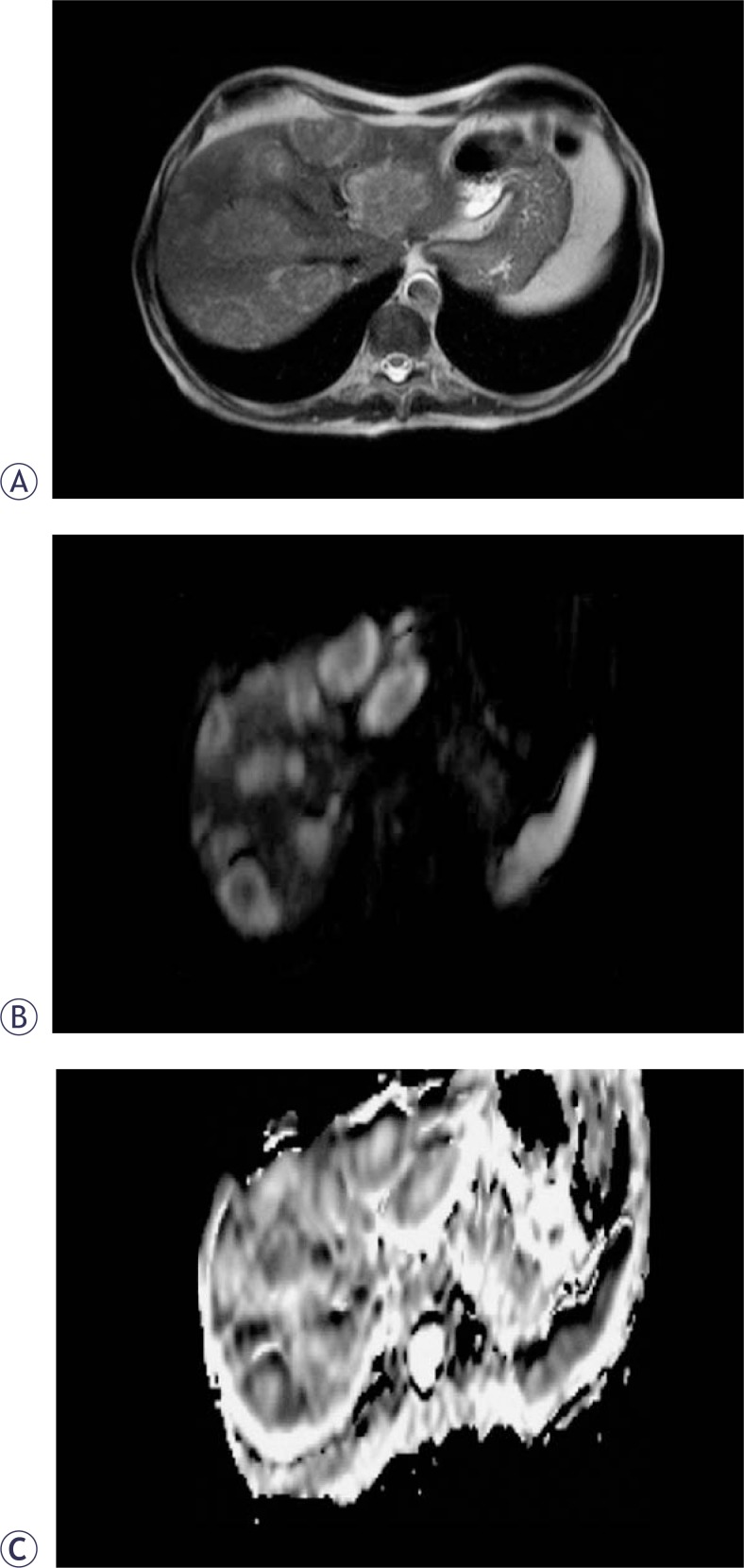
69-year-old woman with multiple metastases of the liver. Axial T2-weighted TSE (A) MR images show multiple metastases in the right and left lobe of the liver. These metastases appear hyperintense compared to the liver on the diffusion-weighted image with b factor 1000 sec/mm^2^ (B). ADC map. Lesion-to-liver ADC ratio=1.5 (C).

**FIGURE 3:**
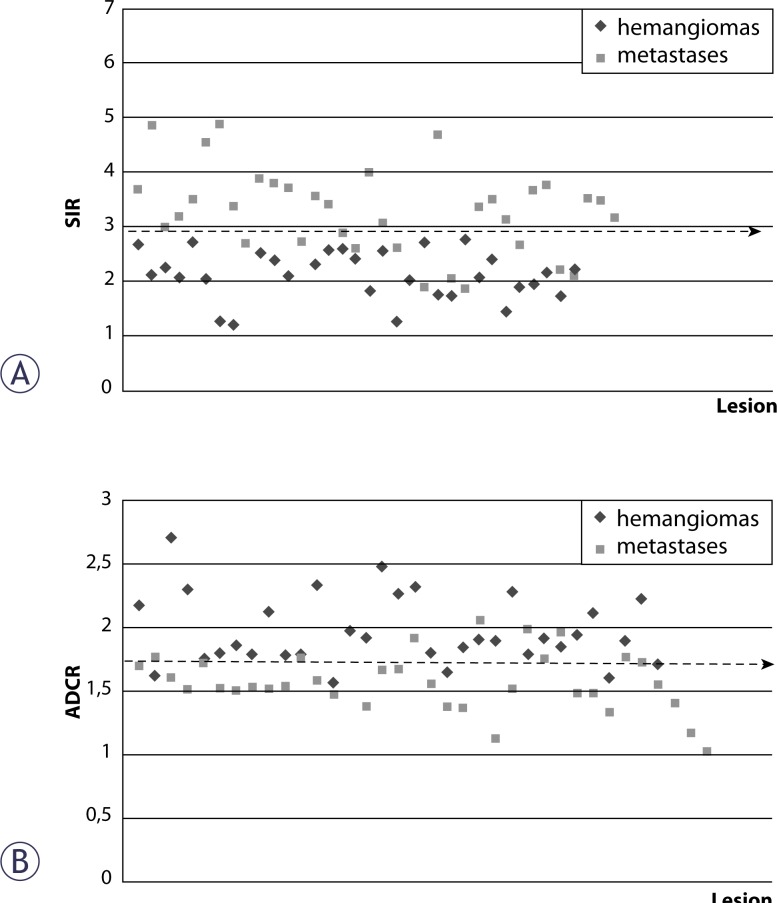
Scattergram distribution of lesion-to-liver SI ratios on DW images with b factor 1000 sec/mm^2^ (A) and ADC ratios (B) of haemangiomas and metastases.

The results of the quantitative analysis of the DW imaging were reviewed in Table 1. With b factors of 0 and 500 sec/mm^2^, no difference of statistical significance was achieved (p > 0.05). With the b factor of 1000 sec/mm^2^, the SIs and SIRs of the metastases were significantly higher than those of the haemangiomas (p < 0.001) (Figures 1B, 2B). The area under the ROC curve was 0.891 ± 0.04 for SIR (p < 0.001). With a cut-off value of 2.6, SIR had a sensitivity of 86% and a specificity of 82% (Figure 3A). The ADCs and ADCRs of metastases were significantly lower than that of the haemangiomas (p < 0.001) (Figures 1C, 2C). The area under the ROC curve was 0.893 ± 0.04 for ADCR (p < 0.001). Setting the cut-off value at 1.7, we found a sensitivity of 88% and a specificity of 72% for ADCR.

## Discussion

For the differential diagnosis of haemangiomas from metastases of the liver, the sensitivity and specificity are generally superior with contrast enhanced MRI when compared to other imaging modalities.^2^ MRI-based techniques are also useful to assess the other hepatic pathology.^7^ However, the greatest clinical experience in the differential diagnosis was with non-specific extracellular gadolinium chelates contrasts because they are safe, relatively inexpensive and they also provide the characterization of most of these lesions.^3^ However, sometimes non-specific extracellular gadolinium chelates may not allow us to recognize these lesions well. In these patients, new contrast agents (SPIO-enhanced MRI) or new MRI techniques (DWI) must be used, especially in patients with the known primary malignancy. In a report published by Nasu *et al.*^8^, the authors compared accuracy of DWI with of SPIO-enhanced MRI in the evaluation of hepatic metastases. In that report it was shown that DWI has more sensitivity than SPIO-enhanced MRI. However, in their study ADC measurement was not performed. In two other reports, the authors compared accuracy of DWI with of SSh T2-W TSE sequences in the evaluation of hepatic metastases. In those reports, although image artifacts were lower with T2-W TSE than SSh-EPI, it is shown that DWI was more useful than SSh T2-W TSE sequences for the detection of lesions.^9^,^10^ However, in daily practice the lesion characterization is as important as the lesion detection.

Recent reports have suggested that DWI with SS EPI may be helpful in the characterization of focal and diffuse liver lesions, with high specificity and sensitivity.^2^,^8^,^11^–^20^ Those studies reported that the ADC values in benign lesions (such as haemangiomas and cysts) were significantly higher than those of the malignant lesions (hepatocellular carcinomas, metastases). This difference was attributed to the difference in cellular density. Since malignant tumors often have higher cellularity than benign lesions, the ADCs of most malignant tumors are lower than benign masses. In these previous studies, different imaging parameters were applied to evaluate a wide range of hepatic lesions, including metastases and haemangiomas. The ADC values of both the normal liver and the liver lesions were differed significantly at different b values.^18^ Namimoto *et al.*^16^ reported a low ADC of the liver with low and high b values (30 and 1200 sec/mm^2^). On the other hand, high ADCs were reported by Taouli *et al.*^15^ using low and intermediate b values (0 and 500 sec/mm^2^). In a study of Yamada *et al*.^18^ in which the b values of 30, 300, 900 or 1100 sec/mm^2^ were used, high ADCs were obtained at low b values. In conclusion, when only a high b value is used, the ADC values reflect the true diffusion of the tissue. On the contrary, when only a low b value is used, the ADC may be influenced by the intravoxel perfusion.^21^

In our study, significant differences between the SIs and SIRs of haemangiomas and metastases were found only on images with a b factor of 1000 sec/mm^2^. At higher b values, the contribution of the T2 shine-through to the signal intensity decreases, while tissue cellularity makes a greater contribution.^22^ Hence, the hyperintensity of metastases on b 1000 sec/mm^2^ images can not be totally attributed to the T2 shine-through effect. Diffusion can be quantitatively evaluated by ADC, which is free of the T2 shine-through effect.^23^ In our series, the mean ADC of the metastases was significantly lower than that of the haemangiomas. Hence, at least a part of the increase in signal on DW images must have been caused by the reduced diffusion in metastases. Since the cavernous haemangiomas are mainly composed of liquid component which consists of fiber septation, scar, and hemorrhage the ADC of the haemangiomas is increased. On the contrary, the metastases have higher cellularity, hence the lower ADC.^1^

This study has several technical limitations. The main limitation was that the SSh-EPI sequence employed with a higher b value had a lower SNR, resulting in greater image distortion. In addition, the EPI sequence caused anatomic distortion due to susceptibility effects.^22^ Although the best lesion conspicuity is achieved with low b value for detecting small focal liver lesions, the best lesion specificity is achieved with a high b value.^22^ Because of that reason, we used a high b value for the characterization of lesions. Another important limitation was that there were not any atypical haemangiomas (such as calcified, hyalinised or sclerosed) and cystic metastases in our study. The necrotic metastasis may exhibit the pronounced hyperintensity on T2-W image and less restricted diffusion.

The differential diagnosis of most of the haemangiomas from metastases is usually possible with the combined use of specific radiologic features. However, sometimes the differential diagnosis of these lesions may still be difficult. Our preliminary data suggest that DWI with a high b value may be helpful in this setting and it can be easily added to routine liver imaging protocols.

## Figures and Tables

**TABLE 1:** Quantitative analysis of DW imaging

	**Metastases (n=36)**	**Haemangiomas (n=33)**	**p**
**SIR (b=1000 sec/mm^2^)**	3.4 ± 0.9	2.2 ± 0.5	< 0.001
**ADC (×10^−3^ mm^2^/sec)**	1.9 ± 0.4	2.5 ± 0.3	< 0.001
**ADCR**	1.6 ± 0.3	1.9 ± 0.3	< 0.001

Note. Data are mean ± SD.

* SIR: lesion-to-liver SI ratio; ADC: apparent diffusion coefficients; ADCR: lesion-to-liver ADC ratio.
